# Supplementary material for: Wolbachia infection in Argentinean populations of Anastrepha fraterculus sp1: preliminary evidence of sex ratio distortion by one of two strains
Source: BMC Microbiol. 2019 Dec 24;19(Suppl 1):289. doi: 10.1186/s12866-019-1652-y (PMC6929328; doi:10.1186/s12866-019-1652-y)
Supplement: Supplementary file 1 — Additional file 1. Symbiont bacteria associated to a male-killing phenotype in arthropods. [file 12866_2019_1652_MOESM1_ESM.pdf]

Additional File 1

Symbiont bacteria associated to a male-killing phenotype in arthropods

Organism	Host	Reference
**Alphaproteobacteria**		
Rickettsia sp.	*Brachys tessellatus*	Lawson et al. 2001. Heredity. 2001;86:497–505.
	Adalia bipunctata	Werren et al. 1994. Insect Mol Biol. 1999;8:133-9.
	Adalia decempunctata	Von der Schulenburg et al. 2001. Appl Environ Microbiol. 2001;67:270-7.
Wolbachia sp.	*Hypolimnas bolina*	Charlat et al. 2006. Curr Biol. 2006 19;16:2453-8.
	Drosophila bifasciata	Hurst et al. 2000. Emer Infect Dis 2000, 6:329-336.
	Acraea encedana	Jiggins et al. 2000. Proc Biol Sci.2000;267:69–73.
**Gammaproteobacteria**		
Arsenophonus nasoniae	*Nasonia vitripennis*	Taylor et al. 2011. J Invertebr Pathol. 2011;106:371-9.
	Bemicia tabaci	Mouton et al. 2012. BMC Microbiol. 2012; 12(Suppl 1): S10.
Bacteroidetes		
Flavobacterium sp.	*Adonia variegata*	Hurst et al. 1999. Parasitol. 1999;118;125-34.

Mollicutes		
Spiroplasma sp.	*Drosophila melanogaster*	Ventura et al. 2012. Microb Ecol. 64:794-801
	Harmonia axyridis	Majerus et al. 1999. Insect Mol Biol. 1999 Nov;8(4):551-5.
