# Supplementary material for: Wolbachia infection in Argentinean populations of Anastrepha fraterculus sp1: preliminary evidence of sex ratio distortion by one of two strains
Source: BMC Microbiol. 2019 Dec 24;19(Suppl 1):289. doi: 10.1186/s12866-019-1652-y (PMC6929328; doi:10.1186/s12866-019-1652-y)
Supplement: Supplementary file 2 — Additional file 2. Nucleotide sequences alignments of Wolbachia genes. Comparison between the partial sequences obtained here (consensus sequences between wAfraCast1_A and wAfraCast2_A) with the most similar sequences from GenBank (NBCI).The genes analyzed are: dnaA (378 bases); aspC (818 b); atpD (881 b); groE (846 b); pdhB (642 b); sucB (612 b) and gltA (554 b). [file 12866_2019_1652_MOESM2_ESM.pdf]

Additional File 2

Nucleotide sequences alignments of *Wolbachia* genes. Comparison between the partial sequences obtained here (consensus sequences between *w*AfraCast1_A and *w*AfraCast2_A) with the most similar sequences from GenBank (NBCI). The genes analyzed are: *dnaA* (378 bases); *aspC* (818 b); *atpD* (881 b)**;** *groE* (846 b); *pdhB* (642 b); *sucB* (612 b) and *gltA* (554 b).

***Wolbachia dnaA* gene**

```
                                     10        20        30        40        50        60        70        80
                             ....|....|....|....|....|....|....|....|....|....|....|....|....|....|....|....|
16514-270-wdnaA.2F378 b      AAGAATAAAATCACGACTCGGTTGGGGGTTGGTGGCAGATATTAATGAAACAACTTTTGAATTAAGGCTTGGTATATTGC
16515-271-wdnaA.2F  378      AAGAATAAAATCACGACTCGGTTGGGGGTTGGTGGCAGATATTAATGAAACAACTTTTGAATTAAGGCTTGGTATATTGC
AE017196.1:153-1535 Wol      AAGAATAAAATCACGACTCGGTTGGGGGTTGGTGGCAGATATTAATGAAACAACTTTTGAATTAAGGCTTGGTATATTGC

                                     90       100       110       120       130       140       150       160
                             ....|....|....|....|....|....|....|....|....|....|....|....|....|....|....|....|
16514-270-wdnaA.2F378 b      AGGCAAAAGTGGAGCAGATGAATATGTATGTTCCGAAAGATGTCCTAGAGTTTTTAGCAAGGAACATAAAATCTAATATA
16515-271-wdnaA.2F  378      AGGCAAAAGTGGAGCAGATGAATATGTATGTTCCGAAAGATGTCCTAGAGTTTTTAGCAAGGAACATAAAATCTAATATA
AE017196.1:153-1535 Wol      AGGCAAAAGTGGAGCAGATGAATATGTATGTTCCGAAAGATGTCCTAGAGTTTTTAGCAAGGAACATAAAATCTAATATA

                                    170       180       190       200       210       220       230       240
                             ....|....|....|....|....|....|....|....|....|....|....|....|....|....|....|....|
16514-270-wdnaA.2F378 b      AGAGAATTAGAAGGAGCATTAAATAAGGTTACCCATACCTCATTAATTGGAAGAAGTATGACGGTAGAATCAGCTAGTGA
16515-271-wdnaA.2F  378      AGAGAATTAGAAGGAGCATTAAATAAGGTTACCCATACCTCATTAATTGGAAGAAGTATGACGGTAGAATCAGCTAGTGA
AE017196.1:153-1535 Wol      AGAGAATTAGAAGGAGCATTAAATAAGGTTACCCATACCTCATTAATTGGAAGAAGTATGACGGTAGAATCAGCTAGTGA

                                    250       260       270       280       290       300       310       320
                             ....|....|....|....|....|....|....|....|....|....|....|....|....|....|....|....|
16514-270-wdnaA.2F378 b      AACCCTAATCGATCTTCTTAGGTCAAATCATAGGTCAGTCACAATAGAAGAAATACAAAAGAAAGTAGCTGAATTTTTCA
16515-271-wdnaA.2F  378      AACCCTAATCGATCTTCTTAGGTCAAATCATAGGTCAGTCACAATAGAAGAAATACAAAAGAAAGTAGCTGAATTTTTCA
AE017196.1:153-1535 Wol      AACCCTAATCGATCTTCTTAGGTCAAATCATAGGTCAGTCACAATAGAAGAAATACAAAAGAAAGTAGCTGAATTTTTCA

                                    330       340       350       360       370
                             ....|....|....|....|....|....|....|....|....|....|....|...
16514-270-wdnaA.2F378 b      ATATAAAGGTTGCAGATATGCAATCCAATAGAAGGCTTCGCAGTCTTGCAAGGCCAAG
16515-271-wdnaA.2F  378      ATATAAAGGTTGCAGATATGCAATCCAATAGAAGGCTTCGCAGTCTTGCAAGGCCAAG
```

```
AE017196.1:153-1535 Wol  ATATAAAGGTTGCAGATATGCAATCCAATAGAAGGCTTCGCAGTCTTGCAAGGCCAAG
```

Wolbachia aspC **gene**

```
                                 10        20        30        40        50        60        70        80
                         ....|....|....|....|....|....|....|....|....|....|....|....|....|....|....|....|
11376-709-aspc.F-cons    GGAGAAcCGGAtTTTGATACTCCaGATCATATAAAAAAAGcaGCTATTCAAGCAATAAGTGAAGGTAAGACTAAATATAC
CP001391.1 Wolbachia sp  GGAGAACCGGATTTTGATACTCCAGATCATATAAAAAAAGCAGCTATTCAAGCAATAAGTGAAGGTAAGACTAAATATAC
DQ235291.1 Wolbachia pi  GGAGAACCGGATTTTGATACTCCAGATCATATAAAAAAAGCAGCTATTCAAGCAATAAGTGAAGGTAAGACTAAATATAC

                                 90       100       110       120       130       140       150       160
                         ....|....|....|....|....|....|....|....|....|....|....|....|....|....|....|....|
11376-709-aspc.F-cons    TGCTGTTGATGGAACGCGTGAGCTGAAAGAAGCAATAATTAATAAGTTAAGAAAGGATAATAACCTCGAGTACACGCTTA
CP001391.1 Wolbachia sp  TGCTGTTGATGGAACGCGTGAGCTGAAAGAAGCAATAATTAATAAGTTAAGAAAGGATAATAACCTCGAGTACACGCTTA
DQ235291.1 Wolbachia pi  TGCTGTTGATGGAACGCGTGAGCTGAAAGAAGCAATAATTAATAAGTTAAGAAAGGATAATAACCTCGAGTACACGCTTA

                                170       180       190       200       210       220       230       240
                         ....|....|....|....|....|....|....|....|....|....|....|....|....|....|....|....|
11376-709-aspc.F-cons    ACCAAATCTGTGTTGGTACAGGTGCTAAGCAGGTGTTATTCAATTTGTTCATGGCAACAATTAACTCTGGAGATGAAGTT
CP001391.1 Wolbachia sp  ACCAAATCTGTGTTGGTACAGGTGCTAAGCAGGTGTTATTCAATTTGTTCATGGCAACAATTAACTCTGGAGATGAAGTT
DQ235291.1 Wolbachia pi  ACCAAATCTGTGTTGGTACAGGTGCTAAGCAGGTGTTATTCAATTTGTTCATGGCAACAATTAACTCTGGAGATGAAGTT

                                250       260       270       280       290       300       310       320
                         ....|....|....|....|....|....|....|....|....|....|....|....|....|....|....|....|
11376-709-aspc.F-cons    ATTATTCCAGCTCCTTATTGGGTTTCATATGTTGATATGGTAAGTCTTTTTGGCGGCTTGCCAGTTGTAGTAGAATGCAA
CP001391.1 Wolbachia sp  ATTATTCCAGCTCCTTATTGGGTTTCATATGTTGATATGGTAAGTCTTTTTGGCGGCTTGCCAGTTGTAGTAGAATGCAA
DQ235291.1 Wolbachia pi  ATTATTCCAGCTCCTTATTGGGTTTCATATGTTGATATGGTAAGTCTTTTTGGCGGCTTGCCAGTTGTAGTAGAATGCAA

                                330       340       350       360       370       380       390       400
                         ....|....|....|....|....|....|....|....|....|....|....|....|....|....|....|....|
11376-709-aspc.F-cons    ACAAAATTTTAAACTGACAGCGGAATTATTGAAAAGCAGGATAACTAAAAAAACTAAATGGTTAATTCTTAACTCACCGA
CP001391.1 Wolbachia sp  ACAAAATTTTAAACTGACAGCGGAATTATTGAAAAGCAGGATAACTAAAAAAACTAAATGGTTAATTCTTAACTCACCGA
DQ235291.1 Wolbachia pi  ACAAAATTTTAAACTGACAGTGGAATTATTGAAAAGCAGGATAACTAAAAAAACTAAATGGTTAATTCTTAACTCACCGA

                                410       420       430       440       450       460       470       480
                         ....|....|....|....|....|....|....|....|....|....|....|....|....|....|....|....|
11376-709-aspc.F-cons    ACAACCCTGCAGGAGCTGTGTACACATGTGATGAATTGAAAGACATAGCACAAATATTGCTTGAATATCCACATATGAAT
CP001391.1 Wolbachia sp  ACAACCCTGCAGGAGCTGTGTACACATGTGATGAATTGAAAGACATAGCACAAATATTGCTTGAATATCCACATATGAAT
```

```
DQ235291.1 Wolbachia pi  ACAACCCTGCAGGAGCTGTGTACACATGTGATGAATTGAAAGACATAGCACAAATATTGCTTGAATATCCACATATGAAT

                                 490       500       510       520       530       540       550       560
                         ....|....|....|....|....|....|....|....|....|....|....|....|....|....|....|....|
11376-709-aspc.F-cons    GTCGTTACAGATGATATTTATGAGCATATAATATACGACGAAAAGTTTTTTACTATCGCTCAGGTTGAGCCGAAGCTTTA
CP001391.1 Wolbachia sp  GTCGTTACAGATGATATTTATGAGCATATAATATACGACGAAAAGTTTTTTACTATCGCTCAGGTTGAGCCGAAGCTTTA
DQ235291.1 Wolbachia pi  GTCGTTACAGATGATATTTATGAGCATATAATATACGACGAAAAGTTTTTTACTATCGCTCAGGTTGAGCCGAAGCTTTA

                                 570       580       590       600       610       620       630       640
                         ....|....|....|....|....|....|....|....|....|....|....|....|....|....|....|....|
11376-709-aspc.F-cons    TGATAGAGTTTTTGTAGTCAATGGAGTATCAAAAGCCTATGCAATGACAGGCTGGAGAATAGGGTATATTGCAGGTAGAA
CP001391.1 Wolbachia sp  TGATAGAGTTTTTGTAGTCAATGGAGTATCAAAAGCCTATGCAATGACAGGCTGGAGAATAGGGTATATTGCAGGTAGAA
DQ235291.1 Wolbachia pi  TGATAGAGTTTTTGTAGTCAATGGAGTATCAAAAGCCTATGCAATGACAGGCTGGAGAATAGGGTATATTGCAGGTAGAA

                                 650       660       670       680       690       700       710       720
                         ....|....|....|....|....|....|....|....|....|....|....|....|....|....|....|....|
11376-709-aspc.F-cons    GTGATGTGGTAAAAGCTATCTCTACGCTGCAGTCTCAAAGCACTTCTAATCCAAATTCGATAGCACAAGCAGCAGCAGCA
CP001391.1 Wolbachia sp  GTGATGTGGTAAAAGCTATCTCTACGCTGCAGTCTCAAAGCACTTCTAATCCAAATTCGATAGCACAAGCAGCAGCAGCA
DQ235291.1 Wolbachia pi  GTGATGTGGTAAAAGCTATCTCTACGCTGCAGTCTCAAAGCACTTCTAATCCAAATTCGATAGCACAAGCAGCAGCAGCA

                                 730       740       750       760       770       780       790       800
                         ....|....|....|....|....|....|....|....|....|....|....|....|....|....|....|....|
11376-709-aspc.F-cons    GCAGCATTAAACGGTGATCATAGTTTTTTGAAAGAAAGAACAAGGATTTTTAAGAGTCGTAGAGATTTTATGGTGAAAGA
CP001391.1 Wolbachia sp  GCAGCATTAAACGGTGATCATAGTTTTTTGAAAGAAAGAACAAGGATTTTTAAGAGTCGTAGAGATTTTATGGTGAAAGA
DQ235291.1 Wolbachia pi  GCAGCATTAAACGGTGATCATAGTTTTTTGAAAGAAAGAACAAGGATTTTTAAGAGTCGTAGAGATTTTATGGTGAAAGA

                                 810
                         ....|....|....|...
11376-709-aspc.F-cons    GCTAAATTCTGCCCCGGG
CP001391.1 Wolbachia sp  GCTAAATTCTGCCCCGGG
DQ235291.1 Wolbachia pi  GCTAAATTCTGCCCCGGG
```

***Wolbachia atpD* gene**

```
                                  10        20        30        40        50        60        70        80
                         ....|....|....|....|....|....|....|....|....|....|....|....|....|....|....|....|
AE017196.1 Wolbachia en  TTGTTGATACAGGTGCACCAATATCGGTGCCAATTGGGCGTTCAACTTTAGGAAGGATTTTTAATGTTGTTGGAGAGCTT
```

```
CP003884.1 Wolbachia en  TTGTTGATACAGGTGCACCAATATCGGTGCCAATTGGGCGTTCAACTTTAGGAAGGATTTTTAATGTTGTTGGAGAGCTT
FJ390351.1 Wolbachia en  TTGTTGATACAGGCGCACCAATATCGGTGCCAATTGGGCGTTCAACTTTAGGAAGGATTTTTAATGTTGTTGGAGAGCTT
CP001391.1 Wolbachia sp  TTGTTGATACAGGTGCACCAATATCGGTGCCAATTGGGCGTTCAACTTTAGGAAGGATTTTTAATGTTGTTGGAGAGCTT
11373-706-atpD.F         TTGTTGAtACaGGTGCAcCaATATCGGTGCCaATTGGGCGTTCaactTTAGGAAGGATTTTTAATGTTGTTGGAGAGCTT

                                 90       100       110       120       130       140       150       160
                         ....|....|....|....|....|....|....|....|....|....|....|....|....|....|....|....|
AE017196.1 Wolbachia en  ATAGATGAGTGTGGTCCACTGAAGGGAAAGTATAACTTAGAGCCTATACACAGAGCACCTCCAAGTTTTACTGAACAGAG
CP003884.1 Wolbachia en  ATAGATGAGTGTGGTCCACTGAAGGGAAAGTATAACTTAGAGCCTATACACAGAGCACCTCCAAGTTTTACTGAACAGAG
FJ390351.1 Wolbachia en  ATAGATGAGTGTGGTCCACTGAAGGGAAAGTATAACTTAGAGCCTATACACAGAGCACCTCCTAGTTTTACTGAACAGAG
CP001391.1 Wolbachia sp  ATAGATGAGTGTGGTCCACTGAAGGGAAAATATAACTTAGAGCCTATACACAGAGCACCTCCAAGTTTTACTGAACAGAG
11373-706-atpD.F         ATAGATGAGTGTGGTCCACTGAAGGGAAAGTATAACTTAGAGCCTATACACAGAGCACCTCCAAGTTTTACTGAACAGAG

                                170       180       190       200       210       220       230       240
                         ....|....|....|....|....|....|....|....|....|....|....|....|....|....|....|....|
AE017196.1 Wolbachia en  AATACAGGAAGAAGTTTTAGTTACGGGAATAAAAGTTATAGATCTTCTTGCACCTTATCTTAAAGGAGGAAAAATTGGCT
CP003884.1 Wolbachia en  AATACAGGAAGAAGTTTTAGTTACGGGAATAAAAGTTATAGATCTTCTTGCACCTTATCTTAAAGGAGGAAAAATTGGCT
FJ390351.1 Wolbachia en  AATACAGGAAGAAGTTTTAGTTACGGGAATAAAAGTTATAGATCTTCTTGCACCTTATCTTAAAGGAGGAAAAATTGGCT
CP001391.1 Wolbachia sp  AATACAGGAAGAAGTTTTAGTTACGGGAATAAAAGTTATAGATCTTCTTGCACCTTATCTTAAAGGAGGAAAAATTGGCT
11373-706-atpD.F         AATACAGGAAGAAGTTTTAGTTACGGGAATAAAAGTTATAGATCTTCTTGCACCTTATCTTAAAGGAGGAAAAATTGGCT

                                250       260       270       280       290       300       310       320
                         ....|....|....|....|....|....|....|....|....|....|....|....|....|....|....|....|
AE017196.1 Wolbachia en  TATTTGGTGGAGCCGGTGTTGGTAAAACAGTCCTAATAATGGAATTAATTAATAATATAGCAAAAGCTCATAAAGGATTT
CP003884.1 Wolbachia en  TATTTGGTGGAGCCGGTGTTGGTAAAACAGTCCTAATAATGGAATTAATTAATAATATAGCAAAAGCTCATAAAGGATTT
FJ390351.1 Wolbachia en  TATTTGGTGGAGCCGGTGTTGGTAAAACAGTCCTAATAATGGAATTAATTAATAATATAGCAAAAGCTCATAAAGGATTT
CP001391.1 Wolbachia sp  TATTTGGTGGAGCCGGTGTTGGTAAAACAGTCCTAATAATGGAATTAATTAATAATATAGCAAAAGCTCATAAAGGATTT
11373-706-atpD.F         TATTTGGTGGAGCCGGTGTTGGTAAAACAGTCCTAATAATGGAATTAATTAATAATATAGCAAAAGCTCATAAAGGGTTT

                                330       340       350       360       370       380       390       400
                         ....|....|....|....|....|....|....|....|....|....|....|....|....|....|....|....|
AE017196.1 Wolbachia en  TCTGTGTTTGCCGGGGTAGGGGAGAGAACGCGTGAAGGTAACGATCTTTATCACGAGATGATCACTTCAAATGTAATAAA
CP003884.1 Wolbachia en  TCTGTGTTTGCCGGGGTAGGGGAGAGAACGCGTGAAGGTAACGATCTTTATCACGAGATGATCACTTCAAATGTAATAAA
FJ390351.1 Wolbachia en  TCTGTGTTTGCCGGGGTAGGGGAGAGAACGCGTGAAGGTAACGATCTTTATCACGAGATGATCACTTCAAATGTAATAAA
CP001391.1 Wolbachia sp  TCTGTGTTTGCCGGGGTAGGGGAGAGAACGCGTGAAGGTAACGATCTTTATCACGAGATGATCACTTCAAATGTAATAAA
11373-706-atpD.F         TCTGTGTTTGCCGGGGTAGGGGAGAGAACGCGTGAAGGTAACGATCTTTATCACGAGATGATCACTTCAAATGTAATAAA
```

```
                                     410       420       430       440       450       460       470       480
                             ....|....|....|....|....|....|....|....|....|....|....|....|....|....|....|....|
AE017196.1 Wolbachia en      TATAAATGAGCATGAAAAATCTCAAGCTGTTTTGGTTTATGGTCAGATGAATGAGCCTCCTGGAGCAAGGGCTAGAGTTG
CP003884.1 Wolbachia en      TATAAATGAGCATGAAAAATCTCAAGCTGTTTTGGTTTATGGTCAGATGAATGAGCCTCCTGGAGCAAGGGCTAGAATTG
FJ390351.1 Wolbachia en      TATAAATGAGCATGAAAAATCTCAAGCTGTTTTGGTTTATGGTCAGATGAATGAGCCTCCTGGAGCAAGGGCTAGAGTTG
CP001391.1 Wolbachia sp      TATAAATGAGCATGAAAAATCTCAAGCTGTTTTGGTTTATGGTCAGATGAATGAGCCTCCTGGAGCAAGGGCTAGAGTTG
11373-706-atpD.F             TATAAATGAGCATGAAAAATCTCAAGCTGTTTTGGTTTATGGTCAGATGAATGAGCCTCCTGGAGCAAGGGCTAGAGTTG

                                     490       500       510       520       530       540       550       560
                             ....|....|....|....|....|....|....|....|....|....|....|....|....|....|....|....|
AE017196.1 Wolbachia en      CTTTAACAGCACTTACTATGGCAGAGTATTTTCGTGACCGTGAAAACCAAGATGTTCTATTTTTTGTGGATAATATCTTT
CP003884.1 Wolbachia en      CTTTAACAGCACTTACTATGGCAGAGTATTTTCGTGACCGTGAAAACCAAGATGTTCTATTTTTTGTGGATAATATCTTT
FJ390351.1 Wolbachia en      CTTTAACAGCACTTACTATGGCAGAGTATTTTCGTGACCGTGAAAACCAAGATGTTCTATTTTTTGTGGATAATATCTTT
CP001391.1 Wolbachia sp      CTTTAACAGCACTTACTATGGCAGAGTATTTTCGTGACCGTGAAAACCAAGATGTTCTATTTTTTGTGGATAATATCTTT
11373-706-atpD.F             CTTTAACAGCACTTACTATGGCAGAGTATTTTCGTGACCGTGAAAACCAAGATGTTCTATTTTTTGTGGATAATATCTTT

                                     570       580       590       600       610       620       630       640
                             ....|....|....|....|....|....|....|....|....|....|....|....|....|....|....|....|
AE017196.1 Wolbachia en      AGATTTACACAAGCTGGTTCTGAAATTTCTGCTTTACTTGGAAGAATACCGTCAGCTGTTGGTTATCAGCCAACCCTTGC
CP003884.1 Wolbachia en      AGATTTACACAAGCTGGTTCTGAAATTTCTGCTTTACTTGGAAGAATACCGTCAGCTGTTGGTTATCAGCCAACCCTTGC
FJ390351.1 Wolbachia en      AGATTTACACAAGCTGGTTCTGAAATTTCTGCTTTACTTGGAAGAATACCGTCAGCTGTTGGTTATCAGCCAACCCTTGC
CP001391.1 Wolbachia sp      AGATTTACACAAGCTGGTTCTGAAATTTCTGCTTTACTTGGAAGAATACCGTCAGCTGTTGGTTATCAGCCAACCCTTGC
11373-706-atpD.F             AGATTTACACAAGCTGGTTCTGAAATTTCTGCTTTACTTGgAAGAATACCGTCAGCTGTTGGTTATCAGCCAACCCTTGC

                                     650       660       670       680       690       700       710       720
                             ....|....|....|....|....|....|....|....|....|....|....|....|....|....|....|....|
AE017196.1 Wolbachia en      AACTGATATGGGTGCAATGCAAGAAAGAATAGCTTCAACAACTTCTGGCTCTATTACTTCTGTGCAAGCTATATATGTTC
CP003884.1 Wolbachia en      AACTGATATGGGTGCAATGCAAGAAAGAATAGCTTCAACAACTTCTGGCTCTATTACTTCTGTGCAAGCTATATATGTTC
FJ390351.1 Wolbachia en      AACTGATATGGGTGCAATGCAAGAAAGAATAGCTTCAACAACTTCTGGCTCTATTACTTCTGTGCAAGCTATATATGTTC
CP001391.1 Wolbachia sp      AACTGATATGGGTGCAATGCAAGAAAGAATAGCTTCAACAACTTCTGGCTCTATTACTTCTGTGCAAGCTATATATGTTC
11373-706-atpD.F             AACTGATATGGGTGCAATGCAAGAAAGAATAGCTTCAACAACTTCTGGCTCTATTACTTCTGTGCAAGCTATATATGTTC

                                     730       740       750       760       770       780       790       800
                             ....|....|....|....|....|....|....|....|....|....|....|....|....|....|....|....|
AE017196.1 Wolbachia en      CTGCGGACGATTTGACTGATCCAGCCCCAGCAACTACATTCTCTCACCTTGATGCAACCACAGTGTTGTCAAGGCAAATA
CP003884.1 Wolbachia en      CTGCGGACGATTTAACTGATCCAGCCCCAGCAACTACATTCTCTCACCTTGATGCAACCACAGTGTTGTCAAGGCAAATA
FJ390351.1 Wolbachia en      CTGCGGACGATTTGACTGATCCAGCCCCAGCAACTACATTCTCTCACCTTGATGCAACCACAGTGTTGTCAAGGCAAATA
```

```
CP001391.1 Wolbachia sp  CTGCGGACGATTTAACTGATCCAGCTCCAGCAACTACGTTCTCTCACCTTGATGCCACCACAGTGTTGTCAAGGCAAATA
11373-706-atpD.F         CTGCGGACGATTTGACTGATCCAGCCCCAGCAACTACATTCTCTCaCCTTGATGCAACCACAGTGTTGTCAAGGCAAATA

                                  810       820       830       840       850       860       870       880
                         ....|....|....|....|....|....|....|....|....|....|....|....|....|....|....|....|
AE017196.1 Wolbachia en  GCTGAAATGGGAATATACCCTGCTGTTGATCCACTTGATTCAACTTCTCAGTCTTTATCTGCTGAAATCATTGGTGAAGA
CP003884.1 Wolbachia en  GCTGAAATGGGAATATACCCTGCTGTTGATCCACTTGATTCAACTTCTCAGTCTTTATCTGCTGAAATCATTGGTGAAGA
FJ390351.1 Wolbachia en  GCTGAAATGGGAATATACCCTGCTGTTGATCCACTTGATTCAACTTCTCAGTCTTTATCTGCTGAAATCATTGGTGAAGA
CP001391.1 Wolbachia sp  GCTGAAATGGGAATATACCCTGCTGTTGATCCACTTGATTCAACTTCTCAGTCTTTATCTGCTGAAATCATTGGTGAAGA
11373-706-atpD.F         GCTGAAATGGGAATATACCCTGCTGTTGATCCACTTGATTCAACTTCTCaGTCTTTATCTGCTGAAATCATTGGTGAAGA


                         .
AE017196.1 Wolbachia en  A
CP003884.1 Wolbachia en  A
FJ390351.1 Wolbachia en  A
CP001391.1 Wolbachia sp  A
11373-706-atpD.F         A
```

Wolbachia groE **gene**

```
                                   10        20        30        40        50        60        70        80
                         ....|....|....|....|....|....|....|....|....|....|....|....|....|....|....|....|
16513-269-wgro.F1        AGCAATAACTGCAGGACCTAGAGGAAAAACAGTAGGGATTAATAAGCCCTATGGAGCACCAGAAATTACAAAAGATGGTT
AY714811.1 Wolbachia en  AGCAATAACTGCAGGACCTAGAGGAAAAACAGTAGGGATTAATAAGCCCTATGGAGCACCAGAAATTACAAAAGATGGTT
AY714809.1 Wolbachia en  AGCAATAACTGCAGGACCTAGAGGAAAAACAGTAGGGATTAATAAGCCCTATGGAGCACCAGAAATTACAAAAGATGGTT
CP011148.1 Wolbachia en  AGCAATAACTGCAGGACCTAGAGGAAAAACAGTAGGGATTAATAAGCCCTATGGAGCACCAGAAATTACAAAAGATGGTT
AE017196.1 Wolbachia en  AGCAATAACTGCAGGACCTAGAGGAAAAACAGTAGGGATTAATAAGCCCTATGGAGCACCAGAAATTACAAAAGATGGTT
CP001391.1 Wolbachia sp  -GCAATAACTGCGGGACCTAGAGGAAAAACAGTAGGGATTAATAAGCCCTATGGAGCACCAGAAATTACAAAAGATGGTT

                                   90       100       110       120       130       140       150       160
                         ....|....|....|....|....|....|....|....|....|....|....|....|....|....|....|....|
16513-269-wgro.F1        ATAAGGTGATGAAGGGTATCAAGCCTGAAAAACCATTAAACGCTGCGATAGCAAGCATCTTTGCACAGAGTTGTTCTCAA
AY714811.1 Wolbachia en  ATAAGGTGATGAAGGGTATCAAGCCTGAAAAACCATTAAACGCTGCGATAGCAAGCATCTTTGCACAGAGTTGTTCTCAA
AY714809.1 Wolbachia en  ATAAGGTGATGAAGGGTATCAAGCCTGAAAAACCATTAAACGCTGCGATAGCAAGCATCTTTGCACANAGTTGTTCTCAA
CP011148.1 Wolbachia en  ATAAGGTGATGAAGGGTATCAAGCCTGAAAAACCATTAAACTCTGCGATAGCAAGCATCTTTGCACAGAGTTGTTCTCAA
AE017196.1 Wolbachia en  ATAAGGTGATGAAGGGTATCAAGCCTGAAAAACCATTAAACGCTGCGATAGCAAGCATCTTTGCACAGAGTTGTTCTCAA
CP001391.1 Wolbachia sp  ATAAGGTGATGAAGGGTATCAAGCCTGAAAAACCATTAAACGCTGCGATAGCAAGCATCTTTGCACAAAGTTGTTCTCAA
```

Con formato: Español (España)

```
                                      170       180       190       200       210       220       230       240
                                  ....|....|....|....|....|....|....|....|....|....|....|....|....|....|....|....|
16513-269-wgro.F1                 TGTAACGATAAAGTTGGTGATGGTGCAACAACGTGCTCAATACTAACTAGCAACATGATAATGGAAGCTTCAAAATCAAT
AY714811.1 Wolbachia en           TGTAACGATAAAGTTGGTGATGGTACAACAACGTGCTCAATACTAACTAGCAACATGATAATGGAAGCTTCAAAATCAAT
AY714809.1 Wolbachia en           TGTAACGATAAAGTTGGTGATGGTACAACAACGTGCTCAATACTAACTAGCAACATGATAATGGAAGCTTCAAAATCAAT
CP011148.1 Wolbachia en           TGTAACGATAAAGTTGGTGATGGTACAACAACGTGCTCAATACTAACTAGCAACATGATAATGGAAGCTTCAAAATCAAT
AE017196.1 Wolbachia en           TGCAACGATAAAGTTGGTGATGGTACAACAACGTGCTCAATACTAACTAGCAACATGATAATGGAAGCTTCAAAATCAAT
CP001391.1 Wolbachia sp           TGTAACGATAAAGTTGGTGATGGTACAACAACGTGCTCAATACTAACTAGCAACATGATAATGGAAGCTTCAAAATCAAT

                                      250       260       270       280       290       300       310       320
                                  ....|....|....|....|....|....|....|....|....|....|....|....|....|....|....|....|
16513-269-wgro.F1                 TGCTGCTGGAAACGATCGTGTTGGTATTAAAAACGGAATACAGAAGGCAAAAGATGTAATATTAAAGGAAATTGCGTCAA
AY714811.1 Wolbachia en           TGCTGCTGGAAACGATCGTGTTGGTATTAAAAACGGAATACAGAAGGCAAAAGATGTAATATTAAAGGAAATTGCGTCAA
AY714809.1 Wolbachia en           TGCTGCTGGAAACGATCGTGTTGGTATTAAAAACGGAATACAGAAGGCAAAAGATGTAATATTAAAGGAAATTGCGTCAA
CP011148.1 Wolbachia en           TGCTGCTGGAAACGATCGTGTTGGTATTAAAAACGGAATACAGAAGGCAAAAGATGTAATATTAAAGGAAATTGCGTCAA
AE017196.1 Wolbachia en           TGCTGCTGGAAACGATCGTGTTGGTATTAAAAACGGAATACAGAAGGCAAAAGATGTAATATTAAAGGAAATTGCGTCAA
CP001391.1 Wolbachia sp           TGCTGCTGGAAACGATCGTGTTGGTATTAAAAACGGAATACAGAAGGCAAAAGATGTAATATTAAAGGAAATTGCGTCAA

                                      330       340       350       360       370       380       390       400
                                  ....|....|....|....|....|....|....|....|....|....|....|....|....|....|....|....|
16513-269-wgro.F1                 TGTCTCGTACAATTTCTCTAGAGAAAATAGACGAAGTGGCACAAGTTGCAATAATCTCTGCAAATGGTGATAAGGATATA
AY714811.1 Wolbachia en           TGTCTCGTACAATTTCTCTAGAGAAAATAGACGAAGTGGCACAAGTTGCAATAATCTCTGCAAATGGTGATAAGGATATA
AY714809.1 Wolbachia en           TGTCTCGTACAATTTCTCTAGAGAAAATAGACGAAGTGGCACAAGTTGCAATAATCTCTGCAAATGGTGATAAGGATATA
CP011148.1 Wolbachia en           TGTCTCGTACAATTTCTCTAGAGAAAATAGACGAAGTGGCACAAGTTGCAATAATCTCTGCAAATGGTGATAAGGATATA
AE017196.1 Wolbachia en           TGTCTCGTACAATTTCTCTAGAGAAAATAGACGAAGTGGCACAAGTTGCAATAATCTCTGCAAATGGTGATAAGGATATA
CP001391.1 Wolbachia sp           TGTCTCGTACAATTTCTCTAGAGAAAATAGACGAAGTGGCACAAGTTGCAATAATCTCTGCAAATGGTGATAAGGATATA

                                      410       420       430       440       450       460       470       480
                                  ....|....|....|....|....|....|....|....|....|....|....|....|....|....|....|....|
16513-269-wgro.F1                 GGTAACAGTATCGCTGATTCCGTGAAAAAAGTTGGAAAAGAGGGTGTAATAACTGTTGAAGAGAGTAAAGGTTCAAAAGA
AY714811.1 Wolbachia en           GGTAACAGTATCGCTGATTCCGTGAAAAAAGTTGGAAAAGAGGGTGTAATAACTGTTGAAGAGAGTAAAGGTTCAAAAGA
AY714809.1 Wolbachia en           GGTAACAGTATCGCTGATTCCGTGAAAAAAGTTGGAAAAGAGGGTGTAATAACTGTTGAAGAGAGTAAAGGTTCAAAAGA
CP011148.1 Wolbachia en           GGTAACAGTATCGCTGATTCCGTGAAAAAAGTTGGAAAAGAGGGTGTAATAACTGTTGAAGAGAGTAAAGGTTCAAAAGA
AE017196.1 Wolbachia en           GGTAACAGTATCGCTGATTCCGTGAAAAAAGTTGGAAAAGAGGGTGTAATAACTGTTGAAGAGAGTAAAGGTTCAAAAGA
CP001391.1 Wolbachia sp           GGTAACAGTATCGCTGATTCCGTGAAAAAAGTTGGAAAAGAGGGTGTAATAACTGTTGAAGAGAGTAAAGGTTCAAAAGA
```

```
                                490       500       510       520       530       540       550       560
                         ....|....|....|....|....|....|....|....|....|....|....|....|....|....|....|....|
16513-269-wgro.F1        GTTAGAAGTTGAGCTGACTACTGGCATGCAATTTGATCGCGGTTATCTCTCTCCGTATTTTATTACAAATAATGAAAAAA
AY714811.1 Wolbachia en  GTTAGAAGTTGAGCTGACTACTGGCATGCAATTTGATCGCGGTTATCTCTCTCCGTATTTTATTACAAATAATGAAAAAA
AY714809.1 Wolbachia en  GTTAGAAGTTGAGCTGACTACTGGCATGCAATTTGATCGCGGTTATCTCTCTCCGTATTTTATTACAAATAATGAAAAAA
CP011148.1 Wolbachia en  GTTAGAAGTTGAGCTGACTACTGGCATGCAATTTGATCGCGGTTATCTCTCTCCGTATTTTATTACAAATAATGAAAAAA
AE017196.1 Wolbachia en  GTTAGAAGTTGAGCTGACTACTGGCATGCAATTTGATCGCGGTTATCTCTCTCCGTATTTTATTACAAATAATGAAAAAA
CP001391.1 Wolbachia sp  GTTAGAAGTTGAGCTGACTACTGGCATGCAATTTGATCGCGGTTATCTCTCTCCGTATTTTATTACAAATAATGAAAAAA

                                570       580       590       600       610       620       630       640
                         ....|....|....|....|....|....|....|....|....|....|....|....|....|....|....|....|
16513-269-wgro.F1        TGATCGTGGAGCTTGATAATCCTTATCTATTAATTACAGAGAAAAAATTAAATATTATTCAACCTTTACTTCCTATTCTT
AY714811.1 Wolbachia en  TGATCGTGGAGCTTGATAATCCTTATCTATTAATTACAGAGAAAAAATTAAATATTATTCAACCTTTACTTCCTATTCTT
AY714809.1 Wolbachia en  TGATCGTGGAGCTTGATAATCCTTATCTATTAATTACAGAGAAAAAATTAAATATTATTCAACCTTTACTTCCTATTCTT
CP011148.1 Wolbachia en  TGATCGTGGAGCTTGATAATCCTTATCTATTAATTACAGAGAAAAAATTAAATATTATTCAACCTTTACTTCCTATTCTT
AE017196.1 Wolbachia en  TGATCGTGGAGCTTGATAATCCTTATCTATTAATTACAGAGAAAAAATTAAATATTATTCAACCTTTACTTCCTATTCTT
CP001391.1 Wolbachia sp  TGATCGTGGAGCTTGATAATCCTTATCTATTAATTACAGAGAAAAAATTAAATATTATTCAACCTTTACTTCCTGTTCTT

                                650       660       670       680       690       700       710       720
                         ....|....|....|....|....|....|....|....|....|....|....|....|....|....|....|....|
16513-269-wgro.F1        GAAGCTATTGTTAAATCTGGTAAACCTTTGGTTATTATTGCAGAGGATATCGAAGGTGAAGCATTAAGCACTTTAGTTAT
AY714811.1 Wolbachia en  GAAGCTATTGTTAAATCTGGTAAACCTTTGGTTATTATTGCAGAGGATATCGAAGGTGAAGCATTAAGCACTTTAGTTAT
AY714809.1 Wolbachia en  GAAGCTATTGTTAAATCTGGTAAACCTTTGGTTATTATTGCAGAGGATATCGAAGGTGAAGCATTAAGCACTTTAGTTAT
CP011148.1 Wolbachia en  GAAGCTATTGTTAAATCTGGTAAACCTTTGGTTATTATTGCAGAGGATATCGAAGGTGAAGCATTAAGCACTTTAGTTAT
AE017196.1 Wolbachia en  GAAGCTATTGTTAAATCTGGTAAACCTTTGGTTATTATTGCAGAGGATATCGAAGGTGAAGCATTAAGCACTTTAGTTAT
CP001391.1 Wolbachia sp  GAAGCTATTGTTAAATCTGGTAAACCTTTGGTTATTATTGCAGAGGATATCGAAGGTGAAGCATTAAGCACTTTAGTTAT

                                730       740       750       760       770       780       790       800
                         ....|....|....|....|....|....|....|....|....|....|....|....|....|....|....|....|
16513-269-wgro.F1        CAATAAATTGCGTGGTGGTTTAAAAGTTGCTGCAGTAAAAGCTCCAGGTTTTGGTGACAGAAGAAAGGAGATGCTCGAAG
AY714811.1 Wolbachia en  CAATAAATTGCGTGGTGGTTTAAAAGTTGCTGCAGTAAAAGCTCCAGGTTTTGGTGACAGAAGAAAGGAGATGCTCGAAG
AY714809.1 Wolbachia en  CAATAAATTGCGTGGTGGTTTAAAAGTTGCTGCAGTAAAAGCTCCAGGTTTTGGTGACAGAAGAAAGGAGATGCTCGAAG
CP011148.1 Wolbachia en  CAATAAATTGCGTGGTGGTTTAAAAGTTGCTGCAGTAAAAGCTCCAGGTTTTGGTGACAGAAGAAAGGAGATGCTCGAAG
AE017196.1 Wolbachia en  CAATAAATTGCGTGGTGGTTTAAAAGTTGCTGCAGTAAAAGCTCCAGGTTTTGGTGACAGAAGAAAGGAGATGCTCGAAG
CP001391.1 Wolbachia sp  CAATAAATTGCGTGGTGGTTTAAAAGTTGCTGCAGTAAAAGCTCCAGGTTTTGGTGACAGAAGAAAGGAGATGCTCGAAG

                                810       820       830       840
```

```
                         ....|....|....|....|....|....|....|....|....|.
16513-269-wgro.F1        ACATAGCAACTTTAACTGGTGCTAAGTACGTCATAAAAGATGAACT
AY714811.1 Wolbachia en  ACATAGCAACTTTAACTGGTGCTAAGTACGTCATAAAAGATGAAC
AY714809.1 Wolbachia en  ACATAGCAACTTTAACTGGTGCTAAGTACGTCATAAAAGATGAAC
CP011148.1 Wolbachia en  ACATAGCAACTTTAACTGGTGCTAAGTACGTCATAAAAGATGAAC
AE017196.1 Wolbachia en  ACATAGCAACTTTAACTGGTGCTAAGTACGTCATAAAAGATGAAC
CP001391.1 Wolbachia sp  ACATAGCAACTTTGACTGGTGCTAAGTACGTCATAAAAGATGAAC
```

***Wolbachia pdhB* gene**

```
                                 10        20        30        40        50        60        70        80
                         ....|....|....|....|....|....|....|....|....|....|....|....|....|....|....|....|
11374-707-pdhB.F         AGAGTAGTTGATACGCCTATTACCGAACATGGATTtGCTGGCCTTGCTGTTGGAGCGGCATTTGCTGGATTAAAGCCAAT
LK055284.1 Wolbachia ge  AGAGTAGTTGATACGCCTATTACCGAACATGGATTTGCTGGCCTTGCTGTTGGAGCGGCATTTGCTGGATTAAAGCCAAT
CP001391.1 Wolbachia sp  AGAGTAGTTGATACGCCTATTACCGAACATGGATTTGCTGGCCTTGCTGTTGGAGCGGCATTTGCTGGATTAAAGCCAAT
DQ235367.1 Wolbachia pi  AGAGTAGTTGATACGCCTATTACCGAACATGGATTTGCTGGCCTTGCTGTTGGAGCGGCATTTGCTGGATTAAAGCCAAT
AE017196.1 Wolbachia en  AGAATAGTTGATACGCCTATTACCGAACATGGATTTGCTGGCCTTGCTGTTGGAGCGGCATTTGCTGGATTAAAGCCAAT

                                 90       100       110       120       130       140       150       160
                         ....|....|....|....|....|....|....|....|....|....|....|....|....|....|....|....|
11374-707-pdhB.F         AGTCGAGTTTATGACTTTTAATTTTTCTATGCAAGCTATTGACCAAATTGTGAATTCCGCAGCAAAAACAAATTATATGT
LK055284.1 Wolbachia ge  AGTCGAGTTTATGACTTTTAATTTTTCTATGCAAGCTATTGACCAAATTGTGAATTCCGCAGCAAAAACAAATTATATGT
CP001391.1 Wolbachia sp  AGTCGAGTTTATGACTTTTAATTTTTCTATGCAAGCTATTGACCAAATTGTGAATTCCGCAGCAAAAACAAATTATATGT
DQ235367.1 Wolbachia pi  AGTCGAGTTTATGACTTTTAATTTTTCTATGCAAGCTATTGACCAAATTGTGAATTCCGCAGCAAAAACAAATTATATGT
AE017196.1 Wolbachia en  AGTCGAGTTTATGACTTTTAATTTTTCTATGCAAGCTATTGACCAAATTGTGAATTCCGCAGCAAAAACAAATTATATGT

                                170       180       190       200       210       220       230       240
                         ....|....|....|....|....|....|....|....|....|....|....|....|....|....|....|....|
11374-707-pdhB.F         CAGGCGGACAACTTGGATGCCCTATAGTATTTCGTGGACCAAATGGCGCTGCAGCAAGAGTTGCTGCACAACATTCTCAA
LK055284.1 Wolbachia ge  CAGGCGGACAACTTGGATGCCCTATAGTATTTCGTGGACCAAATGGCGCTGCAGCAAGAGTTGCTGCACAACATTCTCAA
CP001391.1 Wolbachia sp  CAGGCGGACAACTTGGATGCCCTATAGTATTTCGTGGACCAAATGGCGCTGCAGCAAGAGTTGCTGCACAACATTCTCAA
DQ235367.1 Wolbachia pi  CAGGCGGACAACTTGGATGCCCTATAGTATTTCGTGGACCAAATGGCGCTGCAGCAAGAGTTGCTGCACAACATTCTCAA
AE017196.1 Wolbachia en  CAGGCGGACAACTTGGATGCCCTATAGTATTTCGTGGACCAAATGGCGCTGCAGCAAGAGTTGCTGCACAACATTCTCAA

                                250       260       270       280       290       300       310       320
                         ....|....|....|....|....|....|....|....|....|....|....|....|....|....|....|....|
11374-707-pdhB.F         TGCTTTGCAGCTTGGTATTCGCATATACCGGGGTTAAAAGTAATAGCACCCTATTTTGCCTCAGATTGCAGAGGTCTGCT
```

```
LK055284.1 Wolbachia ge  TGCTTTGCAGCTTGGTATTCGCATATACCGGGGTTAAAAGTAATAGCACCCTATTTTGCCTCAGATTGCAGAGGTCTGCT
CP001391.1 Wolbachia sp  TGCTTTGCAGCTTGGTATTCGCATATACCGGGGTTAAAAGTAATAGCACCCTATTTTGCCTCAGATTGCAGAGGTCTGCT
DQ235367.1 Wolbachia pi  TGCTTTGCAGCTTGGTATTCGCATATACCGGGGTTAAAAGTAATAGCACCCTATTTTGCCTCAGATTGCAGAGGTCTGCT
AE017196.1 Wolbachia en  TGCTTTGCAGCTTGGTATTCGCATATACCGGGGTTAAAAGTAATAGCACCCTATTTTGCCTCAGATTGCAGAGGTCTGCT

                                330       340       350       360       370       380       390       400
                         ....|....|....|....|....|....|....|....|....|....|....|....|....|....|....|....|
11374-707-pdhB.F         TAAAGCTGCAATTCGTGACCCTAATCCGGTAATATTTCTAGAAAACGAAATAGCTTATGGACATGAGCATGAAGTTTCTG
LK055284.1 Wolbachia ge  TAAAGCTGCAATTCGTGACCCTAATCCGGTAATATTTCTAGAAAACGAAATAGCTTATGGACATGAGCATGAAGTTTCTG
CP001391.1 Wolbachia sp  TAAAGCTGCAATTCGTGACCCTAATCCGGTAATATTTCTAGAAAACGAAATAGCTTATGGACATGAGCATGAAGTTTCTG
DQ235367.1 Wolbachia pi  TAAAGCTGCAATTCGTGACCCTAATCCGGTAATATTTCTAGAAAACGAAATAGCTTATGGACATGAGCATGAAGTTTCTG
AE017196.1 Wolbachia en  TAAAGCTGCAATTCGTGACCCTAATCCGGTAATATTTCTAGAAAACGAAATAGCTTATGGACATGAGCATGAAGTTTCTG

                                410       420       430       440       450       460       470       480
                         ....|....|....|....|....|....|....|....|....|....|....|....|....|....|....|....|
11374-707-pdhB.F         ACTCTGAGCTATCAAACAAAGATTATCTACTTGAGATAGGCAAAGCTGCTGTTATACGGGAAGGAAAGGATGTAACTATC
LK055284.1 Wolbachia ge  ACTCTGAGCTATCAAACAAAGATTATCTACTTGAGATAGGCAAAGCTGCTGTTATACGGGAAGGAAAGGATGTAACTATC
CP001391.1 Wolbachia sp  ACTCTGAGCTATCAAACAAAGATTATCTACTTGAGATAGGCAAAGCTGCTGTTATACGGGAAGGAAAGGATGTAACTATC
DQ235367.1 Wolbachia pi  ACTCTGAGCTATCAAACAAAGATTATCTACTTGAGATAGGCAAAGCTGCTGTTATACGGGAAGGAAAGGATGTAACTATC
AE017196.1 Wolbachia en  ACTCTGAGCTATCAAACAAAGATTATCTACTTGAGATAGGCAAAGCTGCTGTTATACGGGAAGGAAAGGATGTAACTATC

                                490       500       510       520       530       540       550       560
                         ....|....|....|....|....|....|....|....|....|....|....|....|....|....|....|....|
11374-707-pdhB.F         ACTGCTTTTTCATTAAAATTAATGGATGCCTTAAATGCAGCAGATTTACTTTCGAGTAAAGGTATAGAAGCTGAAGTTAT
LK055284.1 Wolbachia ge  ACTGCTTTTTCATTAAAATTAATGGATGCCTTAAATGCAGCAGATTTACTTTCGAGTAAAGGTATAGAAGCTGAAGTTAT
CP001391.1 Wolbachia sp  ACTGCTTTTTCATTAAAATTAATGGATGCCTTAAATGCAGCAGATTTACTTTCGAGTGAAGGTATAGAAGCTGAAGTTAT
DQ235367.1 Wolbachia pi  ACTGCTTTTTCATTAAAATTAATGGATGCCTTAAATGCAGCAGATTTACTTTCGAGTGAAGGTATAGAAGCTGAAGTTAT
AE017196.1 Wolbachia en  ACTGCTTTTTCATTAAAATTAATGGATGCCTTAAATGCAGCAGATTTACTTTCGAGTAAAGGTATAGAAGCTGAAGTTAT

                                570       580       590       600       610       620       630       640
                         ....|....|....|....|....|....|....|....|....|....|....|....|....|....|....|....|
11374-707-pdhB.F         TGACCTCAGAACCTTAAGACCACTTGACACTCAAACTGTTATTAACTCTATTCAAAAGACTAATAGGTTAGTTAGTGTAG
LK055284.1 Wolbachia ge  TGACCTCAGAACCTTAAGACCACTTGACACTCAAACTGTTATTAACTCTATTCAGAAGACTAATAGGTTAGTTAGTGTAG
CP001391.1 Wolbachia sp  TGACCTCAGAACCTTAAGACCACTTGACACTCAAACTGTTATTAACTCTATTCAGAAGACTAATAGGTTAGTTAGTGTAG
DQ235367.1 Wolbachia pi  TGACCTCAGAACCTTAAGACCACTTGACACTCAAACTGTTATTAACTCTATTCAGAAGACTAATAGGTTAGTTAGTGTAG
AE017196.1 Wolbachia en  TGACCTCAGAACCTTAAGACCACTTGACACTCAAACTGTTATTAACTCTATTCAGAAGACTAATAGGTTAGTTAGTGTAG
```

```
                              ..
11374-707-pdhB.F              AA
LK055284.1 Wolbachia ge       AA
CP001391.1 Wolbachia sp       AA
DQ235367.1 Wolbachia pi       AA
AE017196.1 Wolbachia en       AA
```

Wolbachia sucB gene

```
                                       10        20        30        40        50        60        70        80
                              ....|....|....|....|....|....|....|....|....|....|....|....|....|....|....|....|
11375-708-sucB.F-cons         ATGaAcaAAGCTGaAcaACCTGCAATAAAACAATATGAATTGCCaAAAAGTGTAgtAAATGGAGAGCAAAGAGAGGAACG
AE017196.1 Wolbachia en       ATGAACAAAGCTGAACAACCTGCAATAAAACAATATGAATTGCCAAAAAGTGTAGTAAATGGAGAGCAAAGAGAGGAACG
LK055284.1 Wolbachia ge       ATGAACAAAGCTGAACAACCTGCAATAAAACAATATGAATTGCCAAAAAGTGTAGTAAATGGAGAGCAAAGAGAGGAACG
AE017196.1 Wolbachia en       ATGAACAAAGCTGAACAACCTGCAATAAAACAATATGAATTGCCAAAAAGTGTAGTAAATGGAGAGCAAAGAGAGGAACG
CP011148.1 Wolbachia en       ATGAACAAAGCTGAACAACCTGCAATAAAACAATATGAATTGCCAAAAAGTGTAGTAAATGGAGAGCAAAGAGAGGAACG
CP003884.1 Wolbachia en       ATGAACAAAGCTGAACAACCTGCAATAAAACAATATGAATTGCCAAAAAGTGTAGTAAGTGGAGAGCAAAGAGAGGAACG
CP001391.1 Wolbachia sp       ATGAACAAAGCTGAACAACCTGCAATAAAACAATATGAATTGCCAAAAAGTGTAGTAAATGGAGAGCAAAGAGAGGAACG

                                       90       100       110       120       130       140       150       160
                              ....|....|....|....|....|....|....|....|....|....|....|....|....|....|....|....|
11375-708-sucB.F-cons         AGTAAAAATGAGCAAAATAAGGCAAGTAATTGCCGCTCGTTTGAAAGCATCGCAAAATACTGCTGCAATACTGACCACGT
AE017196.1 Wolbachia en       AGTAAAAATGAGCAAAATAAGGCAAGTAATTGCCGCTCGTTTGAAAGCATCGCAAAATACTGCTGCAATACTGACCACGT
LK055284.1 Wolbachia ge       AGTAAAAATGAGCAAAATAAGGCAAGTAATTGCCGCTCGTTTGAAAGCATCGCAAAATACTGCTGCAATACTGACCACGT
AE017196.1 Wolbachia en       AGTAAAAATGAGCAAAATAAGGCAAGTAATTGCCGCTCGTTTGAAAGCATCGCAAAATACTGCTGCAATACTGACCACGT
CP011148.1 Wolbachia en       AGTAAAAATGAGCAAAATAAGGCAAGTAATTGCCGCTCGTTTGAAAGCATCGCAAAATACTGCTGCAATACTGACCACAT
CP003884.1 Wolbachia en       AGTAAAAATGAGCAAAATAAGACAAGTAATTGCCGCTCGTTTGAAAGCATCGCAAAATACTGCTGCAATACTGACCACGT
CP001391.1 Wolbachia sp       AGTAAAAATGAGCAAAATAAGGCAAGTAATTGCCGCTCGTTTGAAAGCATCGCAAAATACTGCTGCAATACTGACCACGT

                                      170       180       190       200       210       220       230       240
                              ....|....|....|....|....|....|....|....|....|....|....|....|....|....|....|....|
11375-708-sucB.F-cons         TCAATGAAATTGACATGAAGAATGTCATGGATCTAAGAGCAAAATATAAGGACGCCTTTGAAAAAAAAATATGGAATAAAA
AE017196.1 Wolbachia en       TCAATGAAATTGACATGAAGAATGTCATGGATCTAAGAGCAAAATATAAGGACGCCTTTGAAAAAAAAATATGGAATAAAA
LK055284.1 Wolbachia ge       TCAATGAAATTGACATGAAGAATGTCATGGATCTAAGAGCAAAATATAAGGACGCCTTTGAAAAAAAAATATGGAATAAAA
AE017196.1 Wolbachia en       TCAATGAAATTGACATGAAGAATGTCATGGATCTAAGAGCAAAATATAAGGACGCCTTTGAAAAAAAAATATGGAATAAAA
```

```
CP011148.1 Wolbachia en  TCAATGAAATTGACATGAAGAATGTCATGGATCTAAGAGCAAAATATAAGGACGCCTTTGAAAAAAAATATGGAATAAAA
CP003884.1 Wolbachia en  TCAATGAAATTGACATGAAGAATGTCATGGATCTAAGAGCAAAATATAAGGACGCCTTTGAAAAAAAATATGGAATAAAA
CP001391.1 Wolbachia sp  TCAATGAAATTGACATGAAGAATGTCATGGATCTAAGAACGAAATATAAGGACGCCTTTGAAAAAAAATATGGAATAAAA

                                250       260       270       280       290       300       310       320
                         ....|....|....|....|....|....|....|....|....|....|....|....|....|....|....|....|
11375-708-sucB.F-cons    CTTGGTTTTATGTCGTTTTTTATAAAGGCAGCGGTGCAAGCATTGAAAGAAATTGCTGAAATTAACGCTGAAATCTCAGG
AE017196.1 Wolbachia en  CTTGGTTTTATGTCGTTTTTTATAAAGGCAGCGGTGCAAGCATTGAAAGAAATTGCTGAAATTAACGCTGAAATCTCAGG
LK055284.1 Wolbachia ge  CTTGGTTTTATGTCGTTTTTTATAAAGGCAGCGGTGCAAGCATTGAAAGAAATTGCTGAAATTAACGCTGAAATCTCAGG
AE017196.1 Wolbachia en  CTTGGTTTTATGTCGTTTTTTATAAAGGCAGCGGTGCAAGCATTGAAAGAAATTGCTGAAATTAACGCTGAAATCTCAGG
CP011148.1 Wolbachia en  CTTGGTTTTATGTCGTTTTTTATAAAGGCAGCGGTGCAAGCATTGAAAGAAATTGCTGAAATTAACGCTGAAATCTCAGG
CP003884.1 Wolbachia en  CTTGGTTTTATGTCGTTTTTTATAAAGGCAGCGGTGCAAGCATTGAAAGAAATTGCTGAAATTAACGCTGAAATCTCAGG
CP001391.1 Wolbachia sp  CTTGGTTTTATGTCGTTTTTTATAAAGGCAGCGGTGCAAGCACTGAAAGAGATTCCTGAAATTAACGCTGAAATCTCAGG

                                330       340       350       360       370       380       390       400
                         ....|....|....|....|....|....|....|....|....|....|....|....|....|....|....|....|
11375-708-sucB.F-cons    CGATGAAATCATATATAAACATTACTATGACGTAGGTGTTGCTGTTGGCACCGATAAAGGTCTTGTTGTACCAGTTATTC
AE017196.1 Wolbachia en  CGATGAAATCATATATAAACATTACTATGACGTAGGTGTTGCTGTTGGCACCGATAAAGGTCTTGTTGTACCAGTTATTC
LK055284.1 Wolbachia ge  CGATGAAATCATATATAAACATTACTATGACGTAGGTGTTGCTGTTGGCACCGATAAAGGTCTTGTTGTACCAGTTATTC
AE017196.1 Wolbachia en  CGATGAAATCATATATAAACATTACTATGACGTAGGTGTTGCTGTTGGCACCGATAAAGGTCTTGTTGTACCAGTTATTC
CP011148.1 Wolbachia en  CGATGAAATCATATNTAAACATTACTATGACGTAGGTGTTGCTGTTGGCACCGATAAAGGTCTTGTTGTACCAGTTATTC
CP003884.1 Wolbachia en  CGATGAAATCATATATAAACATTACTATGACGTAGGTGTTGCTGTTGGCACCGATAAAGGTCTTGTTGTACCAGTTATTC
CP001391.1 Wolbachia sp  CGATGAAATCATATATAAACATTACTATGACATAGGTGTTGCTGTTGGCACTGACAAAGGCCTTGTTGTGCCGGTTATTC

                                410       420       430       440       450       460       470       480
                         ....|....|....|....|....|....|....|....|....|....|....|....|....|....|....|....|
11375-708-sucB.F-cons    GGGGTGCCGATCAGATGTCATTTGCAGAAATTGAGTTGACTTTAGTTGCTCTTGGcAAAAAAGCTCGTGAGGGTAAATTA
AE017196.1 Wolbachia en  GGGGTGCCGATCAGATGTCATTTGCAGAAATTGAGTTGACTTTAGTTGCTCTTGGCAAAAAAGCTCGTGAGGGTAAATTA
LK055284.1 Wolbachia ge  GGGGTGCCGATCAGATGTCATTTGCAGAAATTGAGTTGACTTTAGTTGCTCTTGGCAAAAAAGCTCGTGAGGGTAAATTA
AE017196.1 Wolbachia en  GGGGTGCCGATCAGATGTCATTTGCAGAAATTGAGTTGACTTTAGTTGCTCTTGGCAAAAAAGCTCGTGAGGGTAAATTA
CP011148.1 Wolbachia en  GGGGTGCCGATCAGATGTCATTTGCAGAAATTGAGTTGACTTTAGTTGCTCTTGGCAAAAAAGCTCGTGAGGGTAAATTA
CP003884.1 Wolbachia en  GGGGTGCCGATCAGATGTCATTTGCAGAAATTGAGTTGACTTTAGTTGCTCTTGGCAAAAAAGCACGAGAAGGTAAATTA
CP001391.1 Wolbachia sp  GGAGTGCCGATCAGATGTCATTTGCCGAAATTGAATTGACTTTAGTTGCTCTTGGCAAAAAAGCACGAGAAGGTAAATTA

                                490       500       510       520       530       540       550
                         ....|....|....|....|....|....|....|....|....|....|....|....|....|....|....
11375-708-sucB.F-cons    CAAGTATCAgAAATGGAAGGtGCAACATTTACCATCTCAAACGGtGGAGTATACGGTTCACTTCTTtCCaCTCC
```

```
AE017196.1 Wolbachia en  CAAGTATCAGAAATGGAAGGTGCAACATTTACCATCTCAAACGGTGGAGTATACGGTTCACTTCTTTCCACTCC
LK055284.1 Wolbachia ge  CAAGTATCAGAAATGGAAGGTGCAACATTTACCATCTCAAACGGTGGAGTATACGGTTCACTTCTTTCCACTCC
AE017196.1 Wolbachia en  CAAGTATCAGAAATGGAAGGTGCAACATTTACCATCTCAAACGGTGGAGTATACGGTTCACTTCTTTCCACTCC
CP011148.1 Wolbachia en  CAAGTATCAGAAATGGAAGGTGCAACATTTACCATCTCAAACGGTGGAGTATACGGTTCACTTCTTTCCACTCC
CP003884.1 Wolbachia en  CAAGTATCAGAAATGGAAGGTGCAACATTTACCATTTCAAATGGCGGAGTATATGGTTCGCTCCTTTCTACTCC
CP001391.1 Wolbachia sp  CAAGTATCAGAAATGGAAGGTGCAACATTTACCATTTCAAATGGCGGAGTATATGGTTCGCTCCTTTCTACTCC
```

***Wolbachia wglT* gene**

```
                                 10        20        30        40        50        60        70        80
                         ....|....|....|....|....|....|....|....|....|....|....|....|....|....|....|....|
16511-267-wglt.F1-cons   TGATGGAGATGAAGGAGTTCTTAAATATAGGGGACATAATATAGCTGATTTGGCAGAGAATAATAATTTTACTGCTGTGA
NC_002978.6:1100282-110  TGATGGAGATGAAGGAGTTCTTAAATATAGGGGACATAATATAGCTGATTTGGCAGAGAATAATAATTTTACTGCTGTGA
CP011148.1 Wolbachia en  TGATGGAGATGAAGGAGTTCTTAAATATAGGGGACATAATATAGCTGATTTGGCAGAGAATAATAATTTTACTGCTGTGA
CP003884.1 Wolbachia en  TGATGGAGATGAAGGAGTTCTTAAATATAGGGGACATAATATAGCTGATTTGGCAGAGAATAATAATTTTACTGCTGTGA
AE017196.1 Wolbachia en  TGATGGAGATGAAGGAGTTCTTAAATATAGGGGACATAATATAGCTGATTTGGCAGAGAATAATAATTTTACTGCTGTGA
CP001391.1 Wolbachia sp  TGATGGAGATGAAGGAGTTCTTAAATATAGGGGACATAATATAGCTGATTTGGCAGAGAATAATAATTTTACTGCTGTGA

                                 90       100       110       120       130       140       150       160
                         ....|....|....|....|....|....|....|....|....|....|....|....|....|....|....|....|
16511-267-wglt.F1-cons   TTTATTTATTGCTCTATGGTGAATTACCCAGTTCAGAGCAACACAAAAAAATTTCTTCTCAAAATACAAGAATCATCCAAA
NC_002978.6:1100282-110  TTTATTTATTGCTCTATGGTGAATTACCCAGTTCAGAGCAACACAAAAAAATTTCTTCTCAAAATACAAGAATCATCCAAA
CP011148.1 Wolbachia en  TTTATTTATTGCTCTATGGTGAATTACCCAGTTCAGAGCAACACAAAAAAATTTCTTCTCAAAATACAAGAATCATCCAAA
CP003884.1 Wolbachia en  TTTATTTATTGCTCTATGGTGAATTACCCAGTTCAGAGCAACACAAAAAAATTTCTTCTCAAAATACAAGAATCATCCAAA
AE017196.1 Wolbachia en  TTTATTTATTGCTCTATGGTGAATTACCCAGTTCAGAGCAACACAAAAAAATTTCTTCTCAAAATACAAGAATCATCCAAA
CP001391.1 Wolbachia sp  TTTATTTATTGCTCTATGGTGAATTACCCAGTTCAGAGCAACACAAAAAAATTTCTTCTCAAAATACAAGAATCATCCAAA

                                170       180       190       200       210       220       230       240
                         ....|....|....|....|....|....|....|....|....|....|....|....|....|....|....|....|
16511-267-wglt.F1-cons   GTATCAGAGCAAGTTACAAATGTAATTAAAGCATTTCCAAAAACTGCTCACCCTATGTCAATCTTAGTTGCATGTTTTGC
NC_002978.6:1100282-110  GTATCAGAGCAAGTTACAAATGTAATTAAAGCATTTCCAAAAACTGCTCACCCTATGTCAATCTTAGTTGCATGTTTTGC
CP011148.1 Wolbachia en  GTATCAGAGCAAGTTACAAATGTAATTAAAGCATTTCCAAAAACTGCTCACCCTATGTCAATCTTAGTTGCATGTTTTGC
CP003884.1 Wolbachia en  GTATCAGAGCAAGTTACAAATGTAATTAAAGCATTTCCAAAAACTGCTCACCCTATGTCAATCTTAGTTGCATGTTTTGC
AE017196.1 Wolbachia en  GTATCAGAGCAAGTTACAAATGTAATTAAAGCATTTCCAAAAACTGCTCACCCTATGTCAATCTTAGTTGCATGTTTTGC
CP001391.1 Wolbachia sp  GTATCAGAGCAAGTTACAAATGTAATTAAAGCATTTCCAAAAACTGCTCACCCTATGTCAATCTTAGTTGCATGTTTTGC
```

```
                                   250       260       270       280       290       300       310       320
                           ....|....|....|....|....|....|....|....|....|....|....|....|....|....|....|....|
16511-267-wglt.F1-cons     AAGTTTGTCAGCATCTTATCATGAAAAGCATGGCAACAATGTCAATGGTGAAGACCTAGATTTTGGAATTTCTGCAATAG
NC_002978.6:1100282-110    AAGTTTGTCAGCATCTTATCATGAAAAGCATGGCAACAATGTCAATGGTGAAGACCTAGATTTTGGAATTTCTGCAATAG
CP011148.1 Wolbachia en     AAGTTTGTCAGCATCTTATCATGAAAAGCATGGCAACAATGTCAATGGTGAAGACCTAGATTTTGGAATTTCTGCAATAG
CP003884.1 Wolbachia en     AAGTTTGTCAGCATCTTATCATGAAAAGCATGGCAACAATGTCAATGGTGAAGACCTAGATTTTGGAATTTCTGCAATAG
AE017196.1 Wolbachia en     AAGTTTGTCAGCATCTTATCATGAAAAGCATGGCAACAATGTCAATGGTGAAGACCTAGATTTTGGAATTTCTGCAATAG
CP001391.1 Wolbachia sp     AAGTTTGTCAGCATCTTATCATGAAAAGCATGGCAACAATGTCAATGGTGAAGACCTAGATTTTGGAATTTCTGCAATAG

                                   330       340       350       360       370       380       390       400
                           ....|....|....|....|....|....|....|....|....|....|....|....|....|....|....|....|
16511-267-wglt.F1-cons     CGCAAGTTTCCACAATTATTGCAATGATTTATAGGCATATCAACAATCAGGAATTCATAAATGCTAACAATGAATTAAGT
NC_002978.6:1100282-110    CGCAAGTTTCCACAATTATTGCAATGATTTATAGGCATATCAACAATCAGGAATTCATAAATGCTAACAATGAATTAAGT
CP011148.1 Wolbachia en     CGCAAGTTTCCACAATTATTGCAATGATTTATAGGCATATCAACAATCAGGAATTCATAAATGCTAACAATGAATTAAGT
CP003884.1 Wolbachia en     CGCAAGTTTCCACAATTATTGCAATGATTTATAGGCATATCAACAATCAGGAATTCATAAATGCTAACAATGAATTAAGT
AE017196.1 Wolbachia en     CGCAAGTTTCCACAATTATTGCAATGATTTATAGGCATATCAACAATCAGGAATTCATAAATGCTAACAATGAATTAAGT
CP001391.1 Wolbachia sp     CGCAAGTTTCCACAATTATTGCAATGATTTGTAGGCATAACAACAATCAGGAATTCATAAATGCTAACAATGAATTAAGT

                                   410       420       430       440       450       460       470       480
                           ....|....|....|....|....|....|....|....|....|....|....|....|....|....|....|....|
16511-267-wglt.F1-cons     TACAGTGAAAATTTCTTAAAGATGATATTTGGCGATGCTGTTGATAATGATAAAAGCGCCCTTTTTGCAAAAGCTCTGGA
NC_002978.6:1100282-110    TACAGTGAAAATTTCTTAAAGATGATATTTGGCGATGCTGTTGATAATGATAAAAGCGCCCTTTTTGCAAAAGCTCTGGA
CP011148.1 Wolbachia en     TACAGTGAAAATTTCTTAAAGATGATATTTGGCGATGCTGTTGATAATGATAAAAGCGCCCTTTTTGCAAAAGCTCTGGA
CP003884.1 Wolbachia en     TACAGTGAAAATTTCTTAAAGATGATATTTGGCGATGCTGTTGATAATGATAAAAGCGCCCTTTTTGCAAAAGCTCTGGA
AE017196.1 Wolbachia en     TACAGTGAAAATTTCTTAAAGATGATATTTGGCGATGCTGTTGATAATGATAAAAGCGCCCTTTTTGCAAAAGCTCTGGA
CP001391.1 Wolbachia sp     TACAGTGAAAATTTCTTAAAGATGATATTTGGCGATGCTGTTGATAATGATAAAAGCGCCCTTTTTGCAAAAGCTCTGGA

                                   490       500       510       520       530       540       550       560
                           ....|....|....|....|....|....|....|....|....|....|....|....|....|....|....|....|
16511-267-wglt.F1-cons     TAAAATATTTACTCTCCATGCTGATCATGAACAGAATGCTTCTACGGCGGCTGTCAGATTGGTGGGATCGGCTGGTTCTA
NC_002978.6:1100282-110    TAAAATATTTACTCTCCATGCTGATCATGAACAGAATGCTTCTACGGCGGCTGTCAGATTGGTGGGATCGGCTGGTTCTA
CP011148.1 Wolbachia en     TAAAATATTTACTCTCCATGCTGATCATGAACAGAATGCTTCTACGGCGGCTGTCAGATTGGTGGGATCGGCTGGTTCTA
CP003884.1 Wolbachia en     TAAAATATTTACTCTCCATGCTGATCATGAACAGAATGCTTCTACGGCGGCTGTCAGATTGGTGGGATCGGCTGGTTCTA
AE017196.1 Wolbachia en     TAAAATATTTACTCTCCATGCTGATCATGAACAGAATGCTTCTACGGCGGCTGTCAGATTGGTGGGATCGGCTGGTTCTA
CP001391.1 Wolbachia sp     TAAAATATTTACTCTCCATGCTGATCATGAACAGAATGCTTCTACGGCGGCTGTCAGATTGGTGGGATCGGCTGGTTCTA

                                   570       580       590       600       610
```

```
                           ....|....|....|....|....|....|....|....|....|....|..
16511-267-wglt.F1-cons     ATCTGTTTGCAAGCCTCTCTGCAGGAGTTGCTACACTTTGGGGACCAGCACA
NC_002978.6:1100282-110    ATCTGTTTGCAAGCCTCTCTGCAGGAGTTGCTACACTTTGGGGACCAGCACA
CP011148.1 Wolbachia en    ATCTGTTTGCAAGCCTCTCTGCAGGAGTTGCTACACTTTGGGGACCAGCACA
CP003884.1 Wolbachia en    ATCTGTTTGCAAGCCTCTCTGCAGGAGTTGCTACACTTTGGGGACCAGCACA
AE017196.1 Wolbachia en    ATCTGTTTGCAAGCCTCTCTGCAGGAGTTGCTACACTTTGGGGACCAGCACA
CP001391.1 Wolbachia sp    ATCTGTTTGCAAGCCTCTCTGCAGGAGTTGCTACACTTTGGGGACCAGCACA
```
