# Supplementary material for: Wolbachia infection in Argentinean populations of Anastrepha fraterculus sp1: preliminary evidence of sex ratio distortion by one of two strains
Source: BMC Microbiol. 2019 Dec 24;19(Suppl 1):289. doi: 10.1186/s12866-019-1652-y (PMC6929328; doi:10.1186/s12866-019-1652-y)
Supplement: Supplementary file 3 — Additional file 3. Sex ratio analysis of families from each type of cross. [file 12866_2019_1652_MOESM3_ESM.pdf]

Additional File 3

Sex ratio analysis of families from each type of cross

Type of cross	Family ID‡	# males	# females	# adults	Sex ratio
**Af-Cast-1 x Af-Cast-1 P**	3	32	70	102	0,69*
	6	42	61	103	0,59
	9	22	19	41	0,46
	10	12	9	21	0,43
	19	54	49	103	0,48
	22	6	4	10	0,40
	133	48	53	101	0,52
	138	11	14	25	0,56
**Af-Cast-1 x Af-Cast-2 P**	39	1	20	21	0,95*
	40	58	71	129	0,55
	49	31	43	74	0,58
	51	6	7	13	0,54
	52	19	20	39	0,51
	199	70	152	222	0,68*
	200	15	19	34	0,56
**Af-Cast-2 x Af-Cast-1 P**	66	8	9	17	0,53
	69	33	32	65	0,49
	74	5	4	9	0,44
	75	47	36	83	0,43
	77	12	9	21	0,43
	79	9	3	12	0,25

	83	8	8	16	0,50
	84	22	10	32	0,31
	85	21	17	38	0,45
	157	34	48	82	0,59
	159	22	20	42	0,48
	172	18	19	37	0,51
**Af-Cast-2 x Af-Cast-2 P**	91	19	28	47	0,60
	94	157	131	288	0,45
	96	25	7	32	0,22
	99	29	32	61	0,52
	103	22	19	41	0,46
	104	29	26	55	0,47
	105	14	14	28	0,50
	106	9	19	28	0,68
	107	5	11	16	0,69
	108	7	10	17	0,59
	109	38	31	69	0,45
	112	1	2	3	0,67
	113	27	29	56	0,52
	115	7	4	11	0,36
	116	11	3	14	0,21
	117	8	7	15	0,47
	118	13	14	27	0,52
	120	24	17	41	0,41
	232	9	11	20	0,55
	233	35	43	78	0,55

	241	26	23	49	0,47
	261	7	7	14	0,50
**Af-Cast-1 x Af-Cast-1 F**	3	28	34	62	0,55
	6	7	8	15	0,53
	9	37	53	90	0,59
	10	46	47	93	0,51
	19	3	12	15	0,80
	22	9	11	20	0,55
	133	23	32	55	0,58
	138	29	50	79	0,63
**Af-Cast-1 x Af-Cast-2 F**	34	10	10	20	0,50
	39	32	45	77	0,58
	40	11	12	23	0,52
	49	41	76	117	0,65
	51	13	14	27	0,52
	199	19	43	62	0,69
**Af-Cast-2 x Af-Cast-1 F**	69	1	1	2	0,50
	77	26	30	56	0,54
	83	22	24	46	0,52
	84	18	14	32	0,44
	157	30	27	57	0,47
	159	44	44	88	0,50
	79	5	8	13	0,62
	85	8	7	15	0,47
	74	24	46	70	0,66

	75	8	2	10	0,20
**Af-Cast-2 x Af-Cast-2 F**	233	33	26	59	0,44
	91	17	12	29	0,41
	99	9	12	21	0,57
	106	4	3	7	0,43
	120	0	1	1	1,00
	118	8	10	18	0,56
	96	12	10	22	0,45
	105	4	3	7	0,43

‡ only families with viable descendants were considered (>5 pupae); Type of cross: P: Parental crosses (F1 descendants); F: Filial crosses (F2 descendants) ; # males, #females and #adults represent observed number of individuals; Sex ratio was calculated as: # females/#adults; *: $P<0.05$, G-test of goodness of fit. Corrected critical value (after Bonferroni correction) = 0.0006.
