# Supplementary material for: Wolbachia infection in Argentinean populations of Anastrepha fraterculus sp1: preliminary evidence of sex ratio distortion by one of two strains
Source: BMC Microbiol. 2019 Dec 24;19(Suppl 1):289. doi: 10.1186/s12866-019-1652-y (PMC6929328; doi:10.1186/s12866-019-1652-y)

Additional File 4

Molecular Phylogenetic analysis based on concatenated MLST data (2079 bases) by Maximum Likelihood method (Mega5) following the parameters described by Baldo et al. (2006).

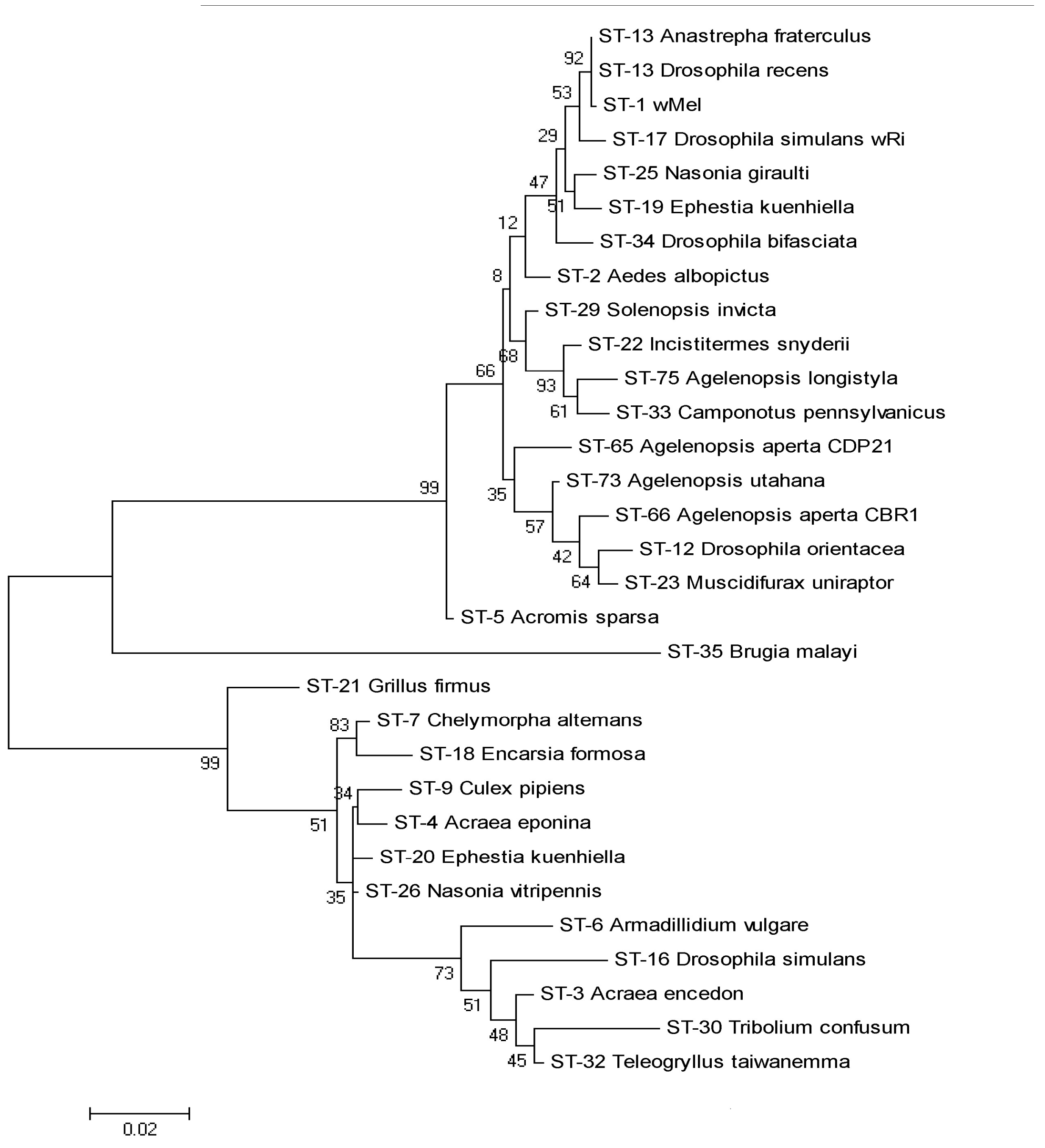

Supplement: Supplementary file 4 — Additional file 4. Molecular Phylogenetic analysis based on concatenated MLST data (2079 bases) by Maximum Likelihood method (Mega5) following the parameters described by Baldo et al. [15]. [file 12866_2019_1652_MOESM4_ESM.pdf]
